# Mutations in Collagen Genes in the Context of an Isolated Population

**DOI:** 10.3390/genes11111377

**Published:** 2020-11-20

**Authors:** Andrej Zupan, Alenka Matjašič, Gašper Grubelnik, Velibor Tasić, Ana Momirovska

**Affiliations:** 1 Institute of Pathology, Faculty of Medicine, University of Ljubljana, Korytkova 2, 1000 Ljubljana, Slovenia; alenka.matjasic@mf.uni-lj.si (A.M.); gasper.grubelnik@mf.uni-lj.si (G.G.); 2 University Children’s Hospital, Mother Theresa 17 St., 1000 Skopje, North Macedonia; vtasic2003@gmail.com; 3 Synlab Laboratories, Pariska 24, 1000 Skopje, North Macedonia; ana.momirovska@synlab.com

**Keywords:** Alport syndrome, benign familial hematuria, digenic inheritance, isolated population, Galičnik

## Abstract

Genetic studies of population isolates have great potential to provide a unique insight into genetic differentiation and phenotypic expressions. Galičnik village is a population isolate located in the northwest region of the Republic of North Macedonia, established around the 10th century. Alport syndrome-linked nephropathy with a complex inheritance pattern has been described historically among individuals in the village. In order to determine the genetic basis of the nephropathies and to characterize the genetic structure of the population, 23 samples were genotyped using a custom-made next generation sequencing panel and 111 samples using population genetic markers. We compared the newly obtained population data with fifteen European population data sets. NGS analysis revealed four different mutations in three different collagen genes in twelve individuals within the Galičnik population. The genetic isolation and small effective population size of Galičnik village have resulted in a high level of genomic homogeneity, with domination of R1a-M458 and R1b-U106* haplogroups. The study explains complex autosomal *in cis* digenic and X-linked inheritance patterns of nephropathy in the isolated population of Galičnik and describes the first case of Alport syndrome family with three different collagen gene mutations.

## 1. Introduction

Studying population isolates in the context of genetic and phenotypic variation can provide a unique insight into genetic differentiation of isolated populations and phenotypic expressions, especially when phenotypically important variants arise either uniquely within that population or begin to exhibit frequency differences across populations. The genetic and phenotypic differentiations between and within populations are often complex and can be the result of a combination of various population effects, such as isolation, selection, migration, bottleneck, adaptation, and genetic drift. Over time, all these effects shape the population genetic structure, making it more homogenous internally and more differentiated from neighboring populations [1,2,3,4].

**Figure 1:**
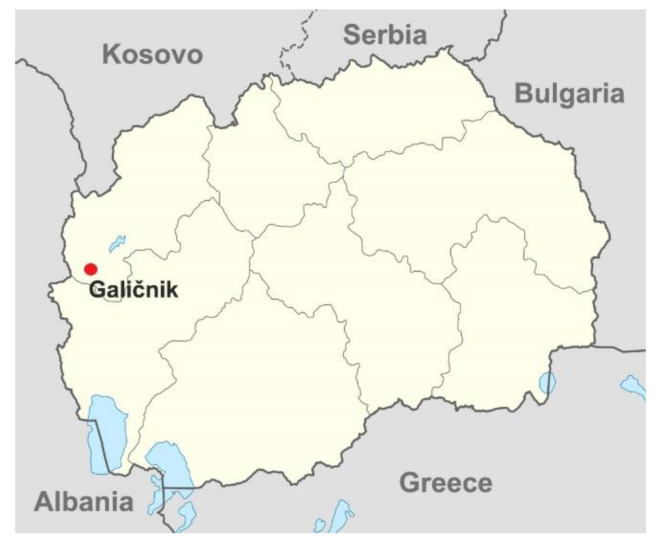
Geographic location of Galičnik village in the Republic of North Macedonia. The map was modified from https://en.wikipedia.org/wiki/Statistical_Regions_of_Macedonia.

Galičnik is an isolated mountainous village in the northwest region of the Republic of North Macedonia (Figure 1). It is one of the two biggest and oldest villages in the region, and was established around the 10th century by a Slavic ethnic group called Mijaks. At the population peak in the 19th century, there were around 1500 individuals in the village. Galičnik village is today almost completely abandoned, although the community of Galičnik still maintains close connections and their heritage through community events. Among the Galičnik population, nephropathy was detected, with clinical manifestations linked to Alport syndrome and/or thin basement membrane nephropathy (benign familial hematuria (BFH)) (OMIM141200). Both nephropathies are an inherited form of basement membrane collagen disorder and are a result of a mutation(s) in genes *COL4A3*, *COL4A4,* and *COL4A5*, which affect the synthesis, assembly, deposition, or function of the collagen IV alpha3, alpha4, and alpha5 molecules. Collagen molecules are the major collagenous constituent of mature mammalian glomerular basement membrane (GBM) [5,6,7]. The most frequent are mutations in the *COL4A5* gene and are the cause of X-linked Alport syndrome (XLAS) (OMIM301050), a semi-dominantly inherited renal disease. XLAS is clinically characterized by severely affected males with persistent hematuria, proteinuria, and commonly hearing loss and ocular anomalies, leading to end-stage renal disease (ESRD), usually during the second or third decade. Females usually have a milder phenotype, with only urinary abnormalities [8]. Mutations in the *COL4A3* and *COL4A4* genes are the cause of autosomal recessive Alport syndrome (ARAS) (OMIM203780), with symptoms similar to those in male XLAS patients and autosomal dominant thin basement membrane disease, with a varying but usually milder phenotype [6,9,10]. Genotype–phenotype correlations have been established, especially in XLAS, with mutations closer to the C-terminal end of the collagen protein, leading to a more severe phenotype [11]. A new form of digenic non-Mendelian inheritance of Alport syndrome, with coexisting mutations in *COL4A3*, *COL4A4,* or *COL4A5* genes, has recently been described, simulating autosomal recessive inheritance (digenic mutations in trans) or autosomal dominant inheritance (mutations in *in cis* mode) [12]. 

The aim of this study was (i) to determine the genetic basis of the nephropathies found among individuals in the Galičnik population, (ii) to assess the influence of population isolation on the genetic structure and inheritance pattern of nephropathies in the Galičnik population, and (iii) to develop a detailed characterization of the genetic structure of the Galičnik population using genetic markers.

## 2. Materials and Methods 

Overall, 111 saliva samples were collected from North Macedonian individuals with a paternal and/or maternal origin from Galičnik village. Informed consent for genetic analysis was obtained from every individual participating in the study. No biopsy tissue nor biopsy results were available. Saliva samples were collected in Oragene-DNA tubes (DNA Genotek, Ottawa, ON, Canada) and genomic DNA was isolated using a QIAamp DNA midi kit (Qiagen, Hilden, Germany). The quantity and quality of DNA samples were determined using a NanoDrop ND-1000 (Thermo Fisher Scientific, Waltham, MA, USA) spectrophotometer. Galičnik samples were subsequently merged with population data from available sources [13,14,15,16,17,18,19,20,21,22,23,24,25,26,27,28,29,30] for a total of 1824 individuals for a biallelic data analysis and 3891 individuals for a Y-STR data analysis, grouped into 15 different European populations.

For the detection of *COL4A3*, *COL4A4*, and *COL4A5* mutations, an AmpliSeq custom-made panel was developed using Ion AmpliSeq designer software (Thermo Fisher Scientific). The assay amplifies all coding exons and flanking regions of the three collagen genes (192 amplicons covering 153 coding exons and flanking regions) in a two-tube multiplex PCR reaction. The final multiplex PCR products were pooled together according to the manufacturer’s instructions and used to prepare a barcoded library suitable for use with an Ion S5 sequencer (Thermo Fisher Scientific). Torrent Suite 2.2 (Thermo Fisher Scientific) software was used to map and align the sequences to the reference sequence (rCRS GenBank sequences NM_000091, NM_000092, and NM_0000495, for *COL4A3*, *COL4A4,* and *COL4A5*, respectively). Variants were detected using Ion Reporter software (Thermo Fisher Scientific) using a custom Ion Reporter filter chain (5000 Exomes and location filter). All filtered variations were visualized and manually inspected using the IGV tool [31,32], and then validated by conventional bidirectional Sanger sequencing on a SeqStudio capillary sequencer (Thermo Fisher Scientific), as described previously [6]. The pathogenicity of variants was determined according to the Clinical Molecular Genetic Society best practice guidelines [33]. The following criteria were selected for pathogenicity determination: literature data, in-frame deletions, non-polymorphic missense mutations involving glycine in the Gly-X-Y triple helical domain, splice-site mutations or truncating mutations. 

For the purposes of Y-chromosome analysis, 44 unrelated male samples were obtained from the Galičnik data set. A total of 16 Y-chromosome biallelic markers were analyzed, following the genealogical hierarchy and considering the recommendations of the Y Chromosome Consortium [34] and the International Society of Genetic Genealogy Haplotype Tree (http://www.isogg.org/tree/). The following set of markers was examined: M215, M78, P15, M198, M434, M458, M334, M343, M269, L23, M412, L11, S116, U152, M126, and U106. The biallelic markers were analyzed using high-resolution melt (HRM) analysis, as described previously [35]. PCR amplification and HRM analysis were performed using a Type-It HRM PCR kit (Qiagen) and carried out on a Rotor Gene-Q instrument (Qiagen), according to the manufacturer’s instructions. The cycling and HRM conditions were chosen according to the manufacturer’s recommendations. Analysis of the HRM results was conducted using Rotor-Gene Q Series software, 2.0.2 (Qiagen).

An AmpFlSTR Yfiler PCR Amplification Kit (Thermo Fisher Scientific) was employed to amplify 17 Y-STR loci (DYS19, DYS389I, DYS389II, DYS390, DYS391, DYS392, DYS393, DYS385a/b, DYS437, DYS438, DYS439, DYS448, DYS456, DYS458, DYS635, and GATA H4) in a single PCR step, according to the manufacturer’s recommendations. The obtained PCR products were loaded onto a SeqStudio capillary sequencer (Thermo Fisher Scientific) and analyzed using GeneMapper 5 software (Thermo Fisher Scientific).

Hypervariable regions I and II (HVR I and HVR II; nucleotide positions 15997–16400 and 30–407) were amplified via PCR, purified, and analyzed through sequencing on a SeqStudio capillary sequencer, in combination with BigDye Terminator v.1.1 chemistry (Thermo Fisher Scientific). Specific haplogroups were determined on the basis of differences from the Cambridge Reference Sequence (rCRS) associated with major Eurasian lineages, and considering the Global Human Mitochondrial DNA (mtDNA) Phylogenetic Tree. 

To visualize the genetic relationships among populations, principal component analysis (PCA) was performed based on the raw frequency data for major haplogroups, using XLSTAT 2018.7 software (2018.7, Addinsoft, Paris, France). Microsatellite-based genetic differences between populations were calculated through analysis of molecular variance (AMOVA) and Rst distance matrices, using Arlequin 3.5 software [36]. The calculations were performed using 1000 permutations for Rst and 20,000 permutations for AMOVA. Arlequin input files were prepared in Microsoft Excel using the Microsat Toolkit Excel plugin. Multidimensional scaling (MDS) analysis was employed as a technique for the two-dimensional representation of dissimilarities among populations, using XLSTAT 2018.7 software. The goodness of fit in MDS analysis was estimated through a stress test. Within specific haplogroups, networks were constructed with Network 4.6.1.1 and Network Publisher 2.0 software (Fluxus Engineering, http://www.fluxus-engineering.com/). The networks were calculated using the median-joining method, with e set to zero, and microsatellite loci weighted according to the estimated variance. 

## 3. Results

### 3.1. Genetic Context of Nephropathy in the Galičnik Population

**Figure 2:**
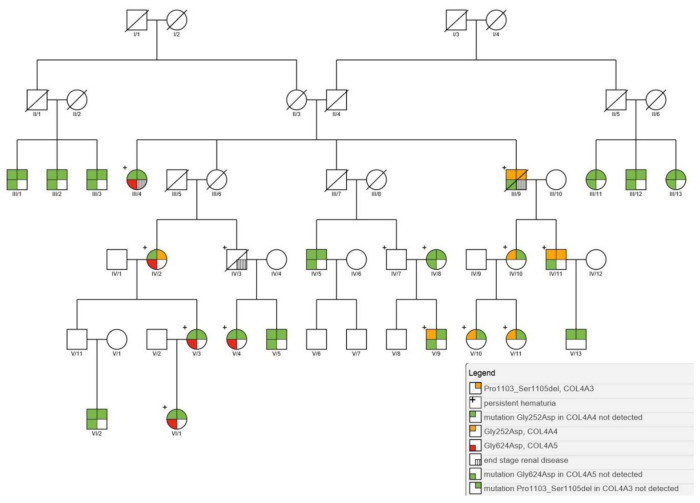
Pedigree tree of the Galičnik family having digenic autosomal inheritance with mutation in *COL4A3* and *COL4A4 in cis* and X-linked *COL4A5* mutation.

Next generation sequencing (NGS) analysis of the collagen genes was performed on affected individuals and their family members to assess the genetic cause of nephropathy. All together, twenty-three individuals of the Galičnik population were genotyped for collagen gene mutations. The analysis revealed four different heterozygous mutations in twelve individuals (10.7%) affecting autosomal chromosome 2 and sex chromosome X. The first mutation was detected in exon 38 of the *COL4A3* gene, resulting in a novel in-frame deletion of three amino acids (c.3307_3315del, p.Pro1103_Ser1105del). The second mutation was detected in exon 13 of the *COL4A4* gene, resulting in a glycine substitution within the Gly-X-Y triple helical domain (c.755G > A, p.Gly252Asp), the third was detected in exon 20 of the *COL4A4* gene, resulting in a large 53 nucleotide deletion (c.1321_1369 + 3del), and the fourth mutation was detected in exon 25 of the *COL4A5* gene, resulting in a glycine substitution in the non-collagenous domain (c.1871G > A, p.Gly624Asp). Novel mutations were submitted to ClinVar (SUB8573868, SUB8573937). Among the four above-mentioned mutations, three were detected within a single family (Figure 2). Segregation analysis revealed (case III/9 to IV/11) that the two autosomal mutations within the large family were inherited together on the same chromosome, like *in cis* (Figure 2). The inheritance pattern resembles a digenic autosomal dominant-like inheritance mode, with a 50% probability of mutation inheritance. Interestingly, four family members (Figure 2) only inherited one autosomal mutation, with patients IV/10, V9, V/10, and V/11 having only the *COL4A4* p.Gly252Asp mutation, and patient IV/2 having a combination of *COL4A3* p.Pro_1103_Ser1105del and *COL4A5* p.Gly624Asp mutations. Detailed results of all family members with clinical data are given in Table 1.

### 3.2. Genetic Context of the Galičnik Population

#### 3.2.1. Y Chromosome

In order to assess Y-chromosome diversity, 44 chromosomes were analyzed for 16 biallelic and 17 STR markers. The vast majority of individuals (56.8%) belonged to haplogroup R1a1a1b1a1 (M458), with the second most common haplogroup being R1b1a1a2 (25%, M269), which further divides into R1b1a1a2a1a1 (13.6%, U106) and R1b1a1a2a1a* (11.4%, U106*). The remaining individuals belonged to haplogroup G2a (11.4%, P15) or haplogroup E1b1b (4.5%, M215).

**Figure 3:**
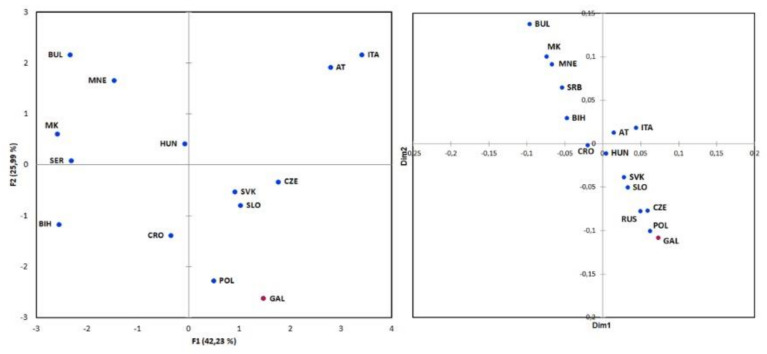
Population structure of the Galičnik samples. Left: Principal component analysis (PCA) of the Galičnik samples with 14 European populations based on raw haplogroups frequencies. Right: Multidimensional scaling (MDS) analysis of the Galičnik samples with 15 European populations based on Rst distance matrix of YSTR haplotypes (AT-Austria, BIH-Bosnia and Herzegovina, BUL-Bulgaria, CRO-Croatia, CZE-Czechia, GAL-Galičnik, HUN-Hungary, ITA-Italia, MK-North Macedonia, MNE-Montenegro, POL-Poland, RUS-Russia, SER-Serbia, SLO-Slovenia, SVK-Slovakia).

To visualize the genetic relationships between populations, we performed principal component analysis on the basis of raw frequencies of biallelic markers of 1824 individuals from 14 European populations (Figure 3). The first two principal components explained 68.2% of the variance, with the Galičnik samples grouping together with the Polish population and close to Slovenian, Croatian, Slovakian, and Czech samples. The Bulgarian, Montenegrin, Macedonian, Serbian, and Bosnian samples formed a separate cluster at the negative pole, while Austrian and Italian samples formed a separate cluster at the positive one. The first principal component (42.2%) represents a north-to-south cline, with the Balkan populations on one side and the Austrian and Italian samples on the opposite side, with the exception of the Galičnik population, which is grouped together with West Slavic and Slovenian samples. The second principal component (26.0%) placed Galičnik and Polish samples apart from other populations at the negative pole, and Bulgarian, Montenegrin, Austrian, and Italian samples at the positive pole (Figure 3).

**Figure 4:**
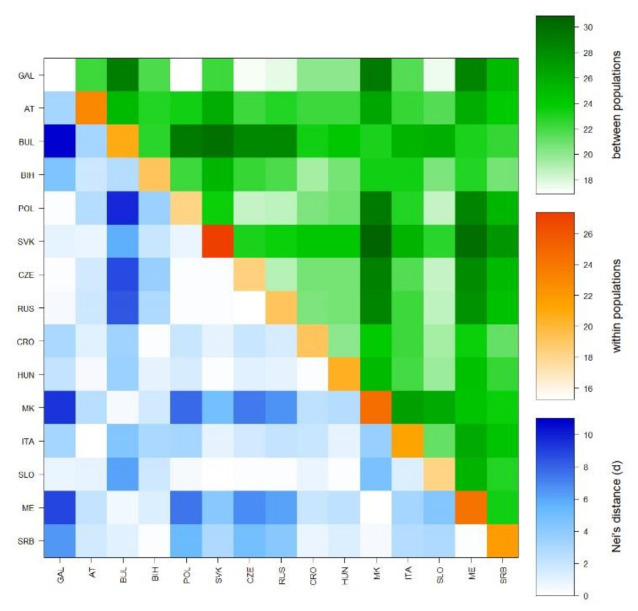
Heatmap based on the average number of pairwise differences (Rst) between populations, within populations, and corrected average pairwise difference.

Relationships between populations were also determined by calculating a pairwise Rst matrix. A heatmap (Figure 4), based on the average number of pairwise differences between populations, within populations, and the corrected average pairwise difference, showed the Galičnik samples clustering together with West Slavic and Slovenian samples and exhibiting the lowest degree of differentiation within the 15 analyzed populations.

Genetic relationships among the 15 populations were further investigated on the basis of Y-STR markers, encompassing 3891 individuals. To visualize genetic differences among populations, an Rst pairwise matrix was constructed and multidimensional scaling (MDS) analysis was performed (Figure 2). The first two dimensions of the MDS plot demonstrated two opposite clusters, with the majority of South Slavic samples on one side of the plot and the West Slavic samples, together with Galičnik and Slovenian samples, on the opposite side, with non-Slavic and Croatian samples in between. Similar to PCA, MDS analysis also revealed the highest similarity between the Galičnik and Polish samples.

**Figure 5:**
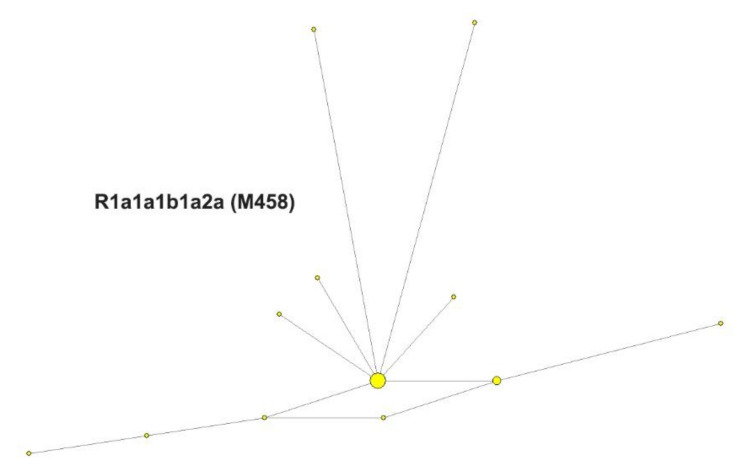
Median-joining network based on 24 Galičnik YSTR haplotypes within the R1a1a (M198) haplogroup. The size of each circle corresponds to the number of individuals belonging to that haplotype. Yellow circles represent haplotypes identified in Galičnik individuals. The lengths of the lines correspond to the number of mutational differences between haplotypes.

To investigate further the haplotype structure within the R1a1a (M198) haplogroup, a median-joining network was constructed (Figure 5), based on 24 Galičnik YSTR haplotypes within R1a1a (M198). The analysis showed a high degree of homogeneity among Galičnik R1a1a haplotypes, with the central haplotype being present in 45.8% of the samples. 

#### 3.2.2. mtDNA

The most common haplogroup detected in the Galičnik population was haplogroup H (28.4%). Further division of haplogroup H revealed four haplogroups in the Galičnik population, with H11 having the highest frequency. Samples that could not be assigned to any of the specific H haplogroups were designated H * haplogroup. The second and third most prevalent haplogroups in the Galičnik population were haplogroups U and N (21.7% and 20.2%, respectively). The frequencies of other detected haplogroups in the Galičnik population are listed in Table 2. 

## 4. Discussion

Centuries of restricted gene flow and endogamy influence the patterns and prevalence of diseases, shaping phenotypes in specific patterns. With the combined effects of endogamy and genetic drift, isolates have been shown to potentially exhibit an increased incidence of recessive disorders [37]. No genetic studies of the isolated Galičnik population have to date been performed.

Our results clearly resolve the complex inheritance pattern for nephropathies in the Galičnik population, revealing four different pathogenic mutations in three different collagen genes. To the best of our knowledge, this is the first study in which three different collagen mutations in three different genes have been described within one family. This unique combination of mutations within different family members is probably a result of the genetic isolation of the community and possible endogamy. It seems that the segregation pattern of the two autosomal mutations in the *COL4A4* (p.Gly252Asp) and *COL4A3* (p.Pro1103_Ser1105del) genes supports the hypothesis of two independent mutations, merged on the same chromosome through recombination events within the small isolated community. This hypothesis is additionally supported by the inheritance pattern, in which we detected only one of the two autosomal mutations in four of the family members (cases IV/2, VI/10, V/9, V/10, and V/11), with the other mutation being lost through a recombination process during transmission from one generation to another. While the *COL4A4* (p.Gly252Asp) mutation has already been described in the literature as a mutation linked to ARAS [38], the *COL4A3* (p.Pro1103_Ser1105del) and *COL4A4* (c.1321_1369 + 3del) mutations were not previously described. We consider both mutations pathogenic, since they result in an in-frame deletion within the Gly-X-Y collagenous domain and a large deletion. Additionally, a similar in-frame deletion in close proximity has already been described in a 32-year-old woman with microhematuria, proteinuria, and short segments of thinning of the GBM and segmental glomerulosclerosis [39]. As shown by clinical data (Table 1), the combination of the two autosomal mutations (cases III/9 and IV/11) *in cis* produces moderately expressed autosomal recessive Alport syndrome. Previous studies have shown that Alport patients with mutations in two different collagen genes usually display a more severe phenotype than those with a single mutation [12]. In our cases, the clinical signs started to manifest in puberty with benign hematuria and proteinuria, slowly developing into the symptoms of Alport syndrome, together with extensive renal impairment and hearing loss at about the age of 45. In the case of proper control of hypertension and other metabolic disturbances, such as hypercholesterolemia and hyperuricemia, life span is not affected, since proper control can lead to stable renal function, thus postponing ESRD. As expected, carriers of only one heterozygotic autosomal mutation in *COL4A4* (p.Gly252Asp) (cases IV/10, V/9, V/10, and V/11) developed benign family hematuria without the development of renal function impairment or hearing loss. In addition to the two detected mutations in autosomal chromosome 2, we also detected a mutation in sex chromosome X within the *COL4A5* gene (p.Gly624Asp) (cases III/4, IV/2, V/3, V/4, and VI/1) within the same family. Among tested individuals, only female carriers with the X-linked *COL4A5* mutation (p.Gly624Asp) were detected (cases III/4, V/3, V/4, and VI/1). The *COL4A5* (p.Gly624Asp) mutation in female cases was manifested by the development of early childhood hematuria with normal hearing, which is consistent with X-linked Alport syndrome in females. The *COL4A5* (p.Gly624Asp) mutation has previously been described in different populations [6,40,41], whereby the authors suggested the possibility of significantly different phenotypes associated with this specific mutation. The *COL4A5* (p.Gly624Asp) mutation is located in the short 12th non-collagenous conserved interruption in exon 25. These interruptions are thought to give natural flexibility to the collagenous structures, and mutations within that region usually cause milder phenotypes than mutations in the Gly-X-Y domain. However, all cases presented in this study carrying the *COL4A5* (p.Gly624Asp) mutation exhibited a remarkably similar phenotype, suggesting modifier genes influencing the phenotype expression between different families as suggested in previous studies [42,43,44]. The complex interaction of disease-causing genes, modifier genes, X-chromosome inactivation, and epigenetic and environmental factors probably plays a significant role in intra- and, more importantly, inter-familial clinical variability, explaining different phenotypes within the same disease-causing mutations. On the other hand, the combination of different mutations can lead to the same or similar phenotypes, as was the case within this study. We would expect a more severe phenotype in the female carrier with the combination of *COL4A5* (p.Gly624Asp) and *COL4A3* (p.Pro1103_Ser1105del) mutations (case IV/2) than in *COL4A5* (p.Gly624Asp)-only mutation carriers. Interestingly, this was not observed in this case, since at the age of 71 only hematuria has been detected, with no hearing loss or ocular changes. Possible mosaicism and/or favorable modifier genes could explain the relatively mild phenotype in this digenic female carrier. Furthermore, a more profound effect of modifier genes in an isolated community is expected, since the modifier allele is more likely to be located on fewer haplotypic backgrounds in a more inbred population, with considerably lower numbers of haplotypes for a specific locus, thereby enhancing its influence. To determine further the complex interaction between disease-causing genes and their modifiers, a further genome-wide association study within Galičnik individuals should be performed.

Y-STR and mtDNA analyses were performed to determine the degree of genetic variability within the Galičnik population. Analysis of genetic variability within the population based on a Y-STR Rst difference matrix (Figure 4) suggests the highest level of genetic homogeneity within the Galičnik population, compared to other populations, which is in agreement with the high level of genetic isolation of the population. The latter was further confirmed by network analysis of the R1a-M458 haplogroup (Figure 5), in which we detected a high level of uniformity among R1a-M458 haplotypes, suggesting a strong founder effect in the population and/or strong drift effect. Analysis of population-wide Y-chromosome variation in Galičnik village showed dominance of haplogroup R1a-M458. Wells et al. [45] suggested that the R1a1a haplogroup spread throughout a considerable part of Europe, with the migration of people manifested archeologically as the Kurgan culture. R1a-M458 is a sub-haplogroup of R1a1a and is the most common Slavic haplogroup among West and East Slavic populations, with the maximum peak in the Polish population, at 30% [19]. This result would be in agreement with the historical events of the Galičnik population since it is believed that Galičnik village was established in the 10th century by a Slavic tribe (Mijak) migrating from the Thessalonica region in Greece. It is known from historical written sources that many Slav tribes settled in the 580s in the region of Thessalonica, which became known as “Macedonian Sclavinia” [46]. The high frequency of haplogroup R1a-M458 could reflect the Slavic origin of the Galičnik population. Furthermore, the high level of the R1a-M458 haplogroup aligns the Galičnik population with other West Slavic populations, as seen by PCA. The especially high level of clustering between the Galičnik and Polish populations can be attributed to the high level of the R1a-M458 haplogroup in both populations. In addition to PCA, MDS analysis based on Y-STR data also highlighted the strong genetic affiliations of the Galičnik population with West Slavic populations, especially Polish, confirming a genetic affiliation on the haplotypic level. The second most common haplogroups in the Galičnik population are R1b-M269 and R1b-U106. In the context of European populations, R1b-M269 reaches maximum frequencies in the eastern area of the Rhine river basin [18]. As described by the Haak et al. [47] study based on ancient DNA samples, both haplogroups R1a and R1b are connected to the arrival of Yamnaya pastoralists from the steppe during the Late Neolithic. In the context of the migration processes of the Galičnik population, R1b and G2a haplogroups may be linked to the older settlers of this area, known as Vlachs or Kuco-vlachs, which refers to the Celtic and Middle East origin of these tribes. Celtic archeological finds have recently been made in the western part of the Republic of North Macedonia, especially around Lake Ohrid [48,49]. Analysis of mtDNA polymorphisms revealed a more heterogenic population structure (Table 2) than shown by Y-STR analysis, which is in line with the global patterns of human mitochondrial DNA and Y-chromosome variation. Because of the widespread phenomenon of patrilocality, it is hypothesized that Y-chromosome variants tend to be more localized geographically than those of mtDNA [50]. The high frequency of haplogroup H is in line with other European populations, with the exceptions of haplogroups N * and HV, setting it apart from the Macedonian population, probably as a result of the small effective population size enhancing the drift effect and fixation of specific haplogroups.

## 5. Conclusions

In conclusion, this is the first study to describe an Alport syndrome family with three different collagen gene mutations within a highly homogenic population structure. The results of classical population genetic markers reflect historical migration and settlements in the Galičnik region.

## Figures and Tables

**Table 1:** Clinical characteristics and corresponding mutations in the Galičnik family.

Case Number	Sex	Age	Hematuria (Age)/Proteinuria	ESRD (Age)	Hearing Loss	Ocular Lesions	Mutation Status		
							COL4A3	COL4A4	COL4A5
II/4	M	deceased (61)	NA	yes (61)	NA	NA	NA	NA	NA
III/4	F	90	yes/no	yes	yes (70)	no	wt	wt	c.1871G > A p.(Gly624Asp)
III/9	M	deceased (80)	yes/yes	yes	yes (70)	no	c.3307_3315del p.(Pro1103_Ser1105del)	c.755G > A p.(Gly252Asp)	wt
IV/2	F	71	yes (EO)/no	no	no	no	c.3307_3315del p.(Pro1103_Ser1105del)	wt	c.1871G > A p.(Gly624Asp)
IV/3	M	deceased (60)	yes (EO)/yes	yes (60)	NA	NA	NA	NA	NA
IV/10	F	53	yes/no	no	no	no	wt	c.755G > A p.(Gly252Asp)	wt
IV/11	F	56	yes (EO)/yes	no	yes	no	c.3307_3315del p.(Pro1103_Ser1105del)	c.755G > A p.(Gly252Asp)	wt
V/3	F	48	yes (EO)/no	no	no	no	wt	wt	c.1871G > A p.(Gly624Asp)
V/4	F	33	yes (EO)/no	no	no	no	wt	wt	c.1871G > A p.(Gly624Asp)
V/9	M	29	yes/no	no	no	no	wt	c.755G > A p.(Gly252Asp)	wt
V/10	F	25	yes (8)/no	no	no	no	wt	c.755G > A p.(Gly252Asp)	wt
V/11	F	21	yes (9)/no	no	no	no	wt	c.755G > A p.(Gly252Asp)	wt
VI/1	F	23	yes (EO)/no	no	no	no	wt	wt	c.1871G > A p.(Gly624Asp)

ESRD, end-stage renal disease; NA, data missing or not available; wt, wild type; EO, early onset.

**Table 2:** mtDNA haplogroup frequencies (%) in the Galičnik population. Lineages defined by the presence of a derived marker and the absence of the derived state at other markers are potentially paraphyletic and under the present nomenclature are differentiated by an asterisk (*), unless they are known to be monophyletic.

**Haplogroup**	%
H	28.4
H *	13.4
H1	4.1
H11	6.8
H17	4.1
HV	18.9
HV8	18.9
U	21.7
U *	1.4
U2	4.1
U3	1.4
U5	
>U5b	2.7
K	
>K *	2.7
>K1	9.4
J1	8.1
J *	1.4
J1c	4.1
J1d	2.6
T	2.7
T1	2.7
N *	20.2
N2 *	1.4
R *	2.7
R0	10.7
W1	5.4
Other	0.0
Population size	74
